# The 10‐year follow‐up of a community‐based cohort of people with diabetes: The incidence of foot ulceration and death

**DOI:** 10.1002/edm2.459

**Published:** 2023-11-21

**Authors:** Shijat Ali Mohammed, Fay Crawford, Genevieve Isabelle Cezard, Michail Papathomas

**Affiliations:** ^1^ School of Mathematics and Statistics University of St Andrews St Andrews UK; ^2^ School of Health and Social Care University of Essex Colchester UK; ^3^ British Heart Foundation Cardiovascular Epidemiology Unit, Department of Public Health and Primary Care University of Cambridge Cambridge UK; ^4^ Victor Phillip Dahdaleh Heart and Lung Research Institute University of Cambridge Cambridge UK

**Keywords:** cohort study, diabetes mellitus, foot ulcer, prediction

## Abstract

**Background:**

Identifying people with diabetes who are likely to experience a foot ulcer is an important part of preventative care. Many cohort studies report predictive models for foot ulcerations and for people with diabetes, but reports of long‐term outcomes are scarce.

**Aim:**

We aimed to develop a predictive model for foot ulceration in diabetes using a range of potential risk factors with a follow‐up of 10 years after recruitment. A new foot ulceration was the outcome of interest and death was the secondary outcome of interest.

**Design:**

A 10‐year follow‐up cohort study.

**Methods:**

1193 people with a diagnosis of diabetes who took part in a study in 2006–2007 were invited to participate in a 10‐year follow‐up. We developed a prognostic model for the incidence of incident foot ulcerations using a survival analysis, Cox proportional hazards model. We also utilised survival analysis Kaplan–Meier curves, and relevant tests, to assess the association between the predictor variables for foot ulceration and death.

**Results:**

At 10‐year follow‐up, 41% of the original study population had died and more than 18% had developed a foot ulcer. The predictive factors for foot ulceration were an inability to feel a 10 g monofilament or vibration from a tuning fork, previous foot ulceration and duration of diabetes.

**Conclusions:**

The prognostic model shows an increased risk of ulceration for those with previous history of foot ulcerations, insensitivity to a 10 g monofilament, a tuning fork and duration of diabetes. The incidence of foot ulceration at 10‐year follow‐up was 18%; however, the risk of death for this community‐based population was far greater than the risk of foot ulceration.

## Graphical abstract

We have conducted a 10‐year follow up of a cohort of people with diabetes who participated in a study of predictive factors for foot ulceration in diabetes in NHS Tayside Scotland. We found the incidence of foot ulceration was 18%, the predictive factors were an inability to feel a 10 g monofilament, the vibrations of a tuning fork, a previous history of foot ulceration and a longer duration of diabetes. Forty one percent of the population died in the intervening 10 years, a lower than average age of survival in Tayside for the same period.

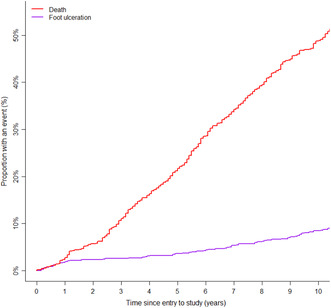

## 1 INTRODUCTION

Data from the International Diabetes Federation (IDF) shows 537 million adults worldwide have a diagnosis of diabetes mellitus in 2022, and this is predicted to rise to 643 million by 2030.^1^ The complications of diabetes can cause premature death and considerable morbidity for people who have a diagnosis of diabetes. Complications affecting the lower limb include vascular and sensory impairment (ischemia and neuropathy) both of which can result in foot ulceration, soft tissue infections and ultimately lower limb amputations. There are high healthcare costs associated with these outcomes and managing complications which affect the lower limb requires the greatest proportion of healthcare expenditure for people with diabetes.^2^

Identifying those who are likely to experience a foot ulcer is an important part of preventative care with national and International Diabetes Clinical Guidelines traditionally recommending that annual foot risk assessments are conducted to categorise a person's risk of developing a foot ulcer as either low/moderate or high.^3^, ^4^

Many cohort studies have been developed to predict the risk of foot ulceration and/or lower limb amputations, and not all have been externally validated.^5^, ^6^, ^7^ Even more rare are reports of patient outcomes followed up over the longer term.

More recently, analyses of patients' routinely collected data have shown the risk of death is considerably higher than that of developing a foot ulcer for people with diabetes and people with diabetes who experience foot ulcers have also been found to have a greater risk of death than those who do not.^8^, ^9^

An author of this manuscript previously published the results of a cohort study conducted between 2006 and 2008 which aimed to quantify the predictive value of elements of clinical history, diagnostic test results and symptoms and signs for foot ulceration in the general diabetes population recruited in a community healthcare setting.^10^ People registered with the NHS Tayside podiatry service in Scotland gave consent for their health data to be collected and analysed (*n* = 1193) and were followed up for an average period of 11 months between 2007 and 2008. The average age of the participants was 70 years, there were almost equal numbers of men and women, and the average duration of diabetes was almost 9 years. These demographic features were comparable to the wider diabetes population in Scotland. Only 23 participants experienced a foot ulcer within the original follow‐up period giving rise to concerns about the accuracy of the model.^10^ As part of a wider research project, we sought additional consent from the participants of the original cohort with diabetes to conduct a long‐term follow‐up of outcomes.^11^

### 1.1 The study aims and objectives

We aimed to develop a predictive model for foot ulceration in diabetes using the incidence of first foot ulcerations 10 years after recruitment. A new foot ulceration was our outcome of interest and death was the secondary outcome.

The primary objectives were to observe the incidence of foot ulceration (outcome) in the cohort of people with diabetes over a 10‐year period and to develop a predictive model for foot ulceration in this group based on 25 explanatory variables collected in the original study considered to be the most readily available from patient records held by interdisciplinary healthcare professionals.^10^, ^12^, ^13^ The secondary objective was to observe the rate of mortality in the cohort population over a 10‐year period. A description of the tests is provided in the Box 1.

BOX 1 **Description of diagnostic tests**

**Ankle brachial index (ABI)**

Patients were in a resting state and their feet level with their hips for at least 20 min before this test was performed. A sphygmomanometer blood pressure gauge (Speider & Keller) was used to measure blood pressure at the arm and ankle. A doppler ultrasound transducer was used to detect a posterior tibial, anterior tibial or brachial pulse. Where the ankle pressures exceeded 220 mmHg blood pressure measurement was abandoned. Ankle pressure was divided by arm pressure to give a ratio, <0.8 was regarded as indicative of ischemia and >1.3 potentially indicative of arterial calcification.

**HbA1c**

Routinely collected data were obtained from an electronic source (Scottish Clinical Information Diabetes Care). Three HbA1c measurements were used to produce an average HbA1c reading for each patient. A reading of 7.5% and less was regarded at target HbA1c and more than 7.5% was regarded as poor blood glucose control.

**Monofilament**

A 10g filament Semmes Weinstein (SWF) was placed at 90° to the foot and pressure applied until the filament bent. Patients were asked if they could feel the touch of the filament on the 1st, 2nd, 4th, 5th met head and apex of the 3rd toe. Inability to feel the touch with a monofilament in either foot was regarded as a positive test result.

**Neurothesiometer**

The voltage was turned up full (50 volts) to allow the patient to feel the vibration on the palm of their hand. The dial was then turned down to zero, the probe was placed against the medial MPJ and the voltage turned up slowly until the patient could feel vibration. This was repeated three times on each side. Vibration readings of >25 volts were considered as a positive test result.

**Neurotip™ (www.owenmumford.com)**

Patients were allowed to feel both the sharp and blunt end of the neurotip on their index finger or the dorsum of the foot before being asked to close their eyes. The blunt end of the neurotip on the plantar aspect of the hallux was always used and patients asked whether they perceive it to be sharp or blunt. Inability to distinguish between sharp and blunt was considered a positive test result.

**Temperature**

The podiatrist placed the two flat end surfaces of a TIP‐Therm® rod on the dorsum of each foot. With their eyes closed patients were asked whether it felt cold or not so cold. Inability to distinguish between cold and not so cold was regarded as a positive test result.

**Tendon hammer**

In a standing position with one knee on a static chair with the Tendo Achilles (TA) clearly visible, the tendon hammer was used to tap the patient's TA. A brisk plantar flexion was judged as a pass and an absent plantar flexion regarded as a positive test result.

**Tuning fork**

An un‐calibrated tuning fork was vibrated on the podiatrist own thigh (to reduce the noise) and placed over the medial aspects the 1st MPJ. Patients were asked whether they could feel the vibration. Inability to feel the vibration was regarded as a positive test result.

**Cotton wool**

Patients were asked to say if they could feel light touch when cotton wool was lightly rubbed over the dorsum of the foot. Inability to feel light touch was regarded as a positive test result.

**Peak plantar pressure**

Peak plantar pressure data were collected using the Pressure Stat system™ manufactured by Podotrack (www.footlogic.info) Patients placed one foot on a single pressure mat. Readings were interpreted by a clinician blind to the results of all other tests and 0–1.5 kg/cm^2^ was the threshold above which (>1.5 to 15 kg/cm^2^) peak plantar pressure was regarded as abnormally high.

## 2 METHODS

### 2.1 Ethics and approvals

Favourable opinions were obtained from Tayside Committee on Medical Research Ethics A (REC number 04/S1401/197), Scotland A Research Ethics Committee [REC reference 16/SS/0213]. Caldicott approval obtained from NHS Tayside [Reference number IGTCAL3842], R&D approval from NHS Fife [Reference 17–01497542]) and BioMed Central ISRCTN clinical trial register [Reference number 10550720].

### 2.2 Participants and consent

The original cohort included 1193 consecutively recruited participants from NHS Tayside community podiatry clinics.^10^ Those with a diagnosis of diabetes mellitus who were ambulant and free of foot ulceration at the time of recruitment gave informed consent and had a detailed examination by one of eight podiatrists to collected baseline explanatory variables (2006 and 2007) (Box 1). The first follow‐up to ascertain the presence of the primary outcome (foot ulceration) was performed on average 11 months after recruitment by podiatrists who scrutinised the hand‐held records of all people who took part for the occurrence of a foot ulcer. It was assumed that the population might receive standard foot care during the conduct of the study but no specific data about this were collected.

For the 10‐year follow‐up study, the subject of this manuscript, participants of the original study were identified via their Community Health Index (CHI) number on an electronic database (SCI Diabetes). Having identified those who had died in the intervening period, a postal letter invited those who survived to participate in the follow‐up study and give consent. Outcome data (foot ulcers) were collected by a podiatrist working in NHS Tayside and ascertained from NHS Tayside patient podiatry records. The NHS podiatrist was unaware of the original risk status of participants as determined in the original cohort study. For the development of the prognostic model, we included information from the entire original cohort. For those that had died in the intervening period and also those that did not respond to a request for consent, we only included their data up to the end of the first 2006–2008 study, for which consent was received. By utilising a Cox proportional hazards model, the absence of follow‐up data due to death or lack of consent was modelled using a right censoring approach.^14^

### 2.3 Sample size

As the size of the sample analysed in this follow‐up study depended on the originally collected dataset and the number of consents, we could only perform a retrospective sample size validity calculation based on the final number of events and model predictors, which can be found in the results section.^15^

### 2.4 Cox proportional hazards model

Cox proportional hazards modelling was used to develop a predictive model for foot ulcerations over a 10‐year follow‐up period (average 129 months). For the modelling purposes, only the first occurrence of foot ulcerations recorded either during the initial or the 10‐year follow‐up period were taken into consideration. We followed participants from their individual date of entry (in 2006–2007) up to a first ulceration, censoring for death and either the end of the initial study period for those who did not provide consent or November 2017 for those who provided additional consent for the 10‐year follow‐up study. Time to event was calculated in months.

The survival analysis was censored for death (*n* = 489) and either the end of the initial study period for those who did not provide consent (*n* = 277) or November 2017 for those who provided additional consent for the 10‐year follow‐up study (*n* = 311). Thus, censoring accounted for the fact that everyone included in the initial study provided consent up to the end of the initial study period. Only those subjects with additional consent for the follow‐up study are followed up to the end of the follow‐up study period.

Univariate Cox proportional hazards models were utilised for all candidate variables in the data set for pre‐selection purposes. Clinical experts were included in discussions about the availability of variables from patients' health records and these discussions also influenced the selection of candidate variables for the multivariable analysis.^7^ No Bonferroni‐type adjustment was made for the significance level of the univariate pre‐selection tests. Although we aimed to reduce the number of variables considered for the initial multivariate model due to sample size and power considerations, we wanted to do so conservatively, pre‐selecting variables even if they were associated with moderate evidence for significance. The multivariate model was developed using a backwards selection algorithm beginning with a model that contained all the significant variables (*p*‐value <0.05) from the univariate analysis. The test of significance for each variable retained in the final multivariate Cox model produced a *p*‐value ≤.05.

To measure the performance of the selected model, the receiver operating characteristic—area under the curve (ROC—AUC) was calculated using the Chambless and Diao's (2006) estimator of AUC for time‐to‐event data.^16^

### 2.5 Competing risk analysis

Competing risk analysis was performed using the cumulative incidence function (CIF) with death as a competing event for foot ulceration.^17^, ^18^

### 2.6 Kaplan–Meier analysis of survival

Further analysis using Kaplan–Meier curves was performed for each binary explanatory variable included in the Cox proportional hazards model, to compare the difference in survival time (all‐cause mortality) between the two groups. The log‐rank test was performed to validate the significance of that difference.

## 3 RESULTS

**FIGURE 1:**
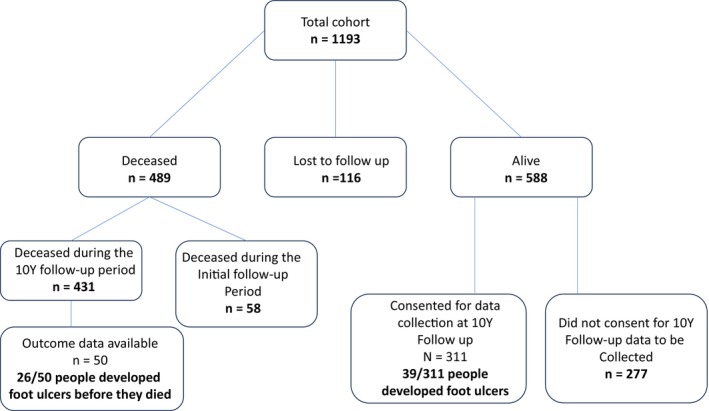
Flow diagram describing the progress of the cohort at the 10‐year follow‐up.

Patient data from the SCI Diabetes database showed that at the 10‐year follow‐up, 489 participants (41%) had died, and 116 participants were lost to follow‐up, leaving 588 participants classified as alive and able to be contacted for their consent (Figure 1). Of the 588 participants who survived, 311 (53%) gave consent for their health records to be accessed. We obtained ethical approval and Caldicott approval from NHS Tayside to ascertain the outcome of 50 deceased participants whose podiatry records were not yet destroyed. Outcome data at 10‐year follow‐up was available for a total of 361 participants, of whom a total of 65 experienced a foot ulceration at 10 years.

Demographic details of people who survived who did not give consent to follow‐up were older, more likely to be female, had diabetes for a shorter duration and fewer ulcers than those who did give consent. (Tables 5 and 6).

The values for the exposure variables collected during the original screening process at the time of recruitment to the study were used as the exposure variables. Table 1 provides the main demographic characteristics of the cohort. Of the 23 participants who developed a foot ulcer after ~1 year in 2008, 16 of those died during the 10‐year follow‐up period.

**TABLE 1:** Characteristics of the cohort at baseline.

Characteristics	Statistics	Value
Number of participants	*N*	1193
Sex		
Females	*N* (%)	581 (48.7%)
Males	*N* (%)	612 (51.3%)
Age (years)	Mean (SD)	70.5 (10.0)
Time from diagnosis of diabetes (years)	Mean (SD)	8.8 (8.4)
Insulin dependency	*N* (%)	276 (23.1%)
Insensitivity to 10 g monofilament	*N* (%)	266 (22.3%)
Absence of pulses	*N* (%)	224 (18.8%)
Insensitivity to tuning fork test	*N* (%)	427 (35.8%)
Abnormal VPT—biothesiometer	*N* (%)	459 (38.5%)
Previous history of ulceration	*N* (%)	82 (6.9%)
Previous history of amputations	*N* (%)	17 (1.4%)
Abnormal pin prick	*N* (%)	586 (49.1%)
Abnormal ankle reflexes	*N* (%)	846 (70.9%)
Unable to record ABI	*N* (%)	223 (18.7%)
Abnormal ABI	*N* (%)	759 (63.6%)
HbA1c	Mean (SD)	7.5 (1.5)
Presence of callus	*N* (%)	326 (27.3%)
Presence of foot deformities	*N* (%)	700 (58.7%)
Not capable of self‐care	*N* (%)	465 (39.0%)
Abnormal peak plantar pressure	*N* (%)	588 (49.3%)
Smoker	*N* (%)	779 (65.3%)
Alcohol consumption	*N* (%)	602 (50.5%)
Living alone	*N* (%)	347 (29.1%)
BMI	Mean (SD)	31.0 (6.0)
Presence of kidney problems	*N* (%)	387 (32.4%)
Insensitivity to temperature	*N* (%)	390 (32.7%)
Presence of eye problems	*N* (%)	192 (16.1%)

*Note*: Recorded at the time of recruitment to original study 2006–2007.

### 3.1 Retrospective sample size calculation

Based on our final Cox model, after backwards selection, we calculated a minimum sample size requirement *n* = 597 for a model with 4 parameters corresponding to the 4 predictor variables. The required number of Events Per Parameter is 11, as calculated by following the approach in Riley et al.^15^ The complete case data set available for developing the multivariate Cox proportional hazards model (with 4 parameters) satisfies the minimum sample size requirements, as it includes observations with information censored due to death or lack of consent after the end of the initial study, that is it includes 1032 observations and 77 foot ulcers (events).^15^

### 3.2 Cox proportional hazards

Table 2 provides the results of univariate Cox proportional hazards models for 26 potential risk factors of foot ulceration. Of the 26 variables tested in the univariate Cox models, we found 14 variables that reached statistical significance at *p* < .05. These variables were also identified during previous discussions with an international group of authors of cohort studies examining the risk of foot ulceration, who considered these explanatory variables to be the easiest to obtain from patient records and therefore possess good clinical utility.^7^

**TABLE 2:** Exploratory variables included in the univariate analysis with foot ulceration as the outcome variable.

Parameter	*n* (complete cases)f	HR (95%CI)	*p*‐value
Sex=‘Women’ (yes vs. no)	1169	0.433 (0.271, 0.693)	<.001
Insulin dependency (yes vs. no)	1169	2.408 (1.555, 3.730)	<.001
Time from diagnosis of diabetes (years)	1167	1.041 (1.023, 1.060)	<.001
Insensitivity to 10 g monofilament (yes vs. no)	1156	4.630 (2.963, 7.233)	<.001
Absence of pulses (yes vs. no)	1169	2.477 (1.570, 3.907)	<.001
Insensitivity to tuning fork test (yes vs. no)	1169	3.336 (2.142, 5.197)	<.001
Abnormal VPT – biothesiometer (yes vs. no)	1169	2.466 (1.596, 3.808)	<.001
Previous history of ulceration (yes vs. no)	1169	3.570 (2.039, 6.249)	<.001
Previous history of amputations (yes vs. no)	1169	6.321 (2.299, 17.38)	<.001
Abnormal pin prick (yes vs. no)	1169	1.805 (1.159, 2.810)	.009
Abnormal ankle reflexes (yes vs. no)	1169	0.549 (0.350, 0.862)	.009
Abnormal ABI (unable to record/missing vs. no)	1169	2.928 (1.302, 6.585)	.009
Abnormal ABI (yes vs. no)	1169	1.525 (0.725, 3.209)	.266
HbA1c (numeric)	1045	1.157 (1.031, 1.298)	.013
Presence of callus (yes vs. no)	1169	0.523 (0.303, 0.904)	.020
Presence of foot deformities (yes vs. no)	1169	1.579 (0.998, 2.500)	.051
Not capable of self‐care (yes vs. no)	1169	1.323 (0.853, 2.050)	.211
Age (years)	1169	1.015 (0.991, 1.039)	.215
Abnormal peak plantar pressure (yes vs. no)	1061	1.331 (0.837, 2.118)	.227
Smoker (yes vs. no)	1169	0.696 (0.372, 1.305)	.259
Alcohol consumption (yes vs. no)	1169	1.166 (0.758, 1.795)	.484
Living alone (yes vs. no)	1169	1.185 (0.736, 1.906)	.485
BMI (numeric)	1058	0.988 (0.947, 1.030)	.563
Presence of kidney problems (yes vs. no)	1026	1.129 (0.713, 1.788)	.606
Insensitivity to temperature (yes vs. no)	1169	1.090 (0.688, 1.726)	.714
Presence of eye problems (yes vs. no)	1169	1.103 (0.610, 1.995)	.745

*Note*: Statistical significance denoted by *p* < .05.

Abbreviation: HR, Hazard Ratio.

The complete case data set available for multivariate analysis of the 14 variables included 1032 individuals with an average duration of diabetes of 8.79 years (SD: 8.12), 507 (49.13%) females and 77‐foot ulcerations. The final multivariate Cox proportional hazards model included: duration of diabetes (years) [Hazard Ratio (HR): 1.039, 95% CI: 1.019–1.058], insensitivity to a 10‐g monofilament [HR: 2.739, 95% CI: 1.673–4.484], in inability to feel a tuning fork [HR: 2.287, 95% CI: 1.409–3.712] and previous history of foot ulceration [HR: 2.564, 95% CI: 1.404–4.682] (Table 3). The fitted model is,

$$\begin{array}{c} \log\frac{h_{i}\left(t\right)}{h_{0}\left(t\right)}=0.038\times \left(\mathrm{Dur}.\right)+1.008\times \left(\text{In}.\mathrm{Mon}.\right)\\ +0.827\times \left(\text{In}.\mathrm{Tun}.\;\text{Fork}\right)+0.941\times \left(\text{Hist}.\right), \end{array}$$

where the value for the last 3 covariates is 1 when there is insensitivity to a 10‐g monofilament, inability to feel a tuning fork, and previous history of ulceration. Otherwise, it is 0. Also, *h*_*i*_ (*t*), is the hazard function, that is the estimated probability that subject *i* experiences an ulceration at time *t* + 1, conditional on the fact that they have not experienced one at time *t*. Additional information on the model fit relevant to prediction is given in the Appendix S1.^19^ The summarised ROC—AUC at 10 years for the final Cox proportional hazards model was 0.732 (95% CI: 0.674–0.8).^18^ This shows an increased risk of ulceration for those with previous history of foot ulcerations, as well as insensitivity to monofilament and tuning fork. The risk for ulceration also increases with diabetic duration.

**TABLE 3:** Multivariate Cox proportional hazards model results.

Parameter	HR (95% CI)	*p*‐value
Time from diagnosis of diabetes (years)	1.039 (1.019, 1.058)	<.001
Insensitivity to 10 g monofilament (yes vs. no)	2.739 (1.673, 4.484)	<.001
Insensitivity to tuning fork test (yes vs. no)	2.287 (1.409, 3.712)	<.001
Previous history of ulceration (yes vs. no)	2.564 (1.404, 4.682)	.002
*Baseline cumulative hazard at time t = 120 months is 0.039*		

*Note*: Statistical significance denoted by *p* < .05.

Abbreviation: HR, Hazard Ratio.

### 3.3 Competing risk analysis

**FIGURE 2:**
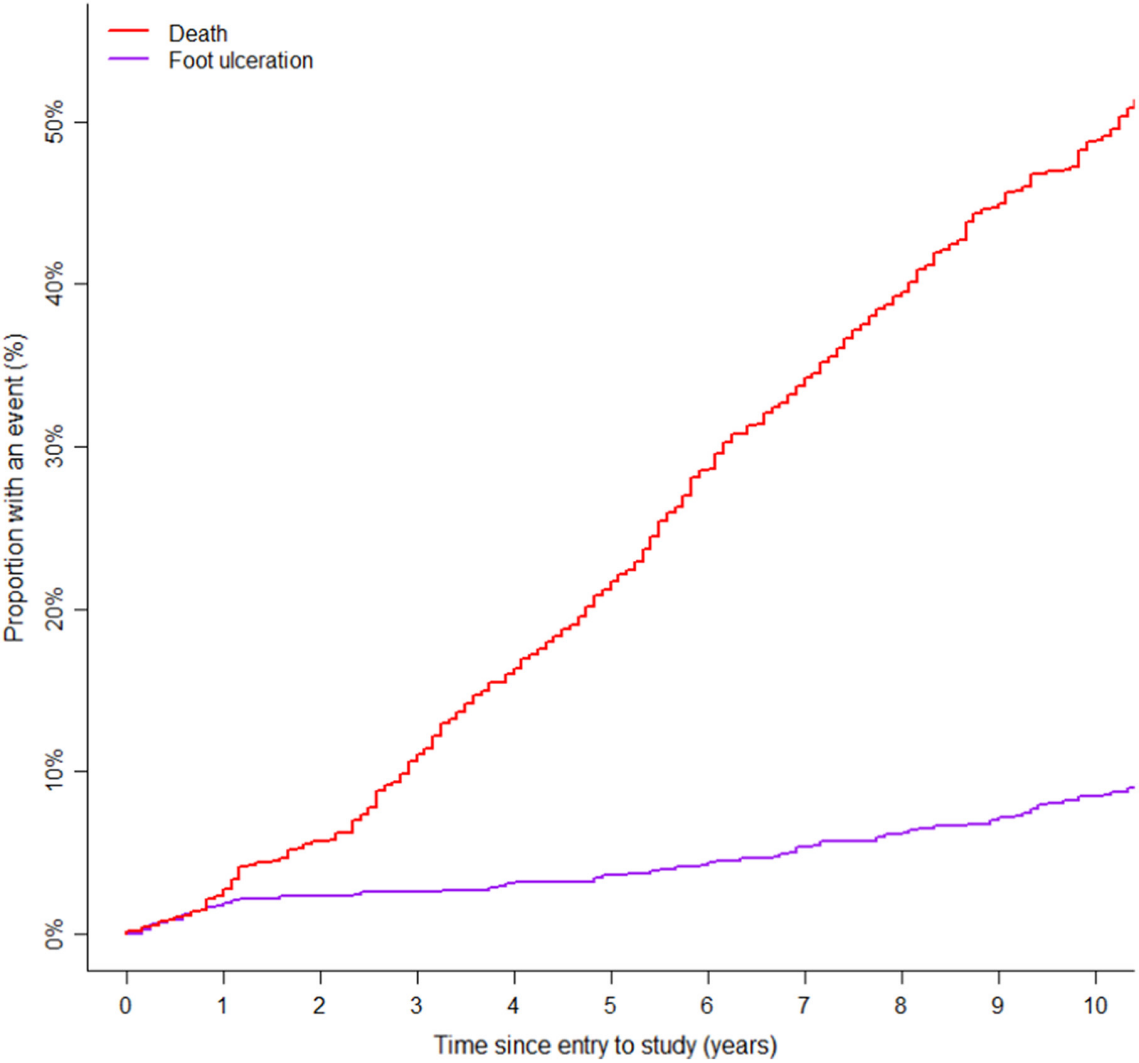
Competing risk analysis: cumulative incidence of death and foot ulcers.

The cumulative incidence of death was calculated as a competing event for foot ulceration. At 10 years, the cumulative incidence for death was 48.87% (95% CI: 48.84, 48.91) and the cumulative incidence for foot ulcers is 8.46% (95% CI: 8.44, 8.48). Figure 2 shows the plot of cumulative incidence. (Figure 2).

Table 4 provides descriptive statistics for the exploratory variables selected in the multivariate model by all‐cause mortality status of the participants at 10‐year follow‐up. For those who exhibit Insensitivity to either 10 g monofilament or VPT tuning fork, we observed a higher percentage with a ‘Dead’ status compared to the ‘Alive’ percentage. Both *p*‐values from the corresponding chi‐square tests of association for the two 2 × 2 cross‐tabulations are <10^−6^, a significant result that shows evidence of an association between Alive/Dead status and Sensitivity/Insensitivity to monofilament or VPT tuning fork. There is also a statistically significant association between Ulceration History and Alive/Dead status (*p*‐value = .04).

**TABLE 4:** Variables within the final multivariate model for alive vs. dead participants.

Label	Complete (*n*)	Missing (*n*)	Levels	Mean (SD)/*N* (%)	
				Alive	Dead
				*n* = 704 (59.0%)	*n* = 489 (41.0%)
Insensitivity to 10 g monofilament	1180	13	No	574 (62.8%)	340 (37.2%)
			Yes	120 (45.1%)	146 (54.9%)
			(Missing)	10 (76.9%)	3 (23.1%)
Time from diagnosis of diabetes (in years)	1191	2	Mean (SD)	8.3 (8.0)	9.6 (8.9)
Insensitivity to VPT tuning fork	1193	0	No	496 (64.8%)	270 (35.2%)
			Yes	208 (48.7%)	219 (51.3%)
Previous history of ulcerations	1193	0	No	665 (59.9%)	446 (40.1%)
			Yes	39 (47.6%)	43 (52.4%)

*Note*: ‘Dead’ relates to all‐cause mortality: cross‐tabulation between Insensitivity to Monofilament and Alive/Dead status, between Insensitivity to VPT tuning fork and Alive/Dead status and between Previous History of Ulceration and Alive/Dead status. Also, mean (standard deviation) of Time from diagnosis of diabetes for Alive vs. Dead participants.

**FIGURE 3:**
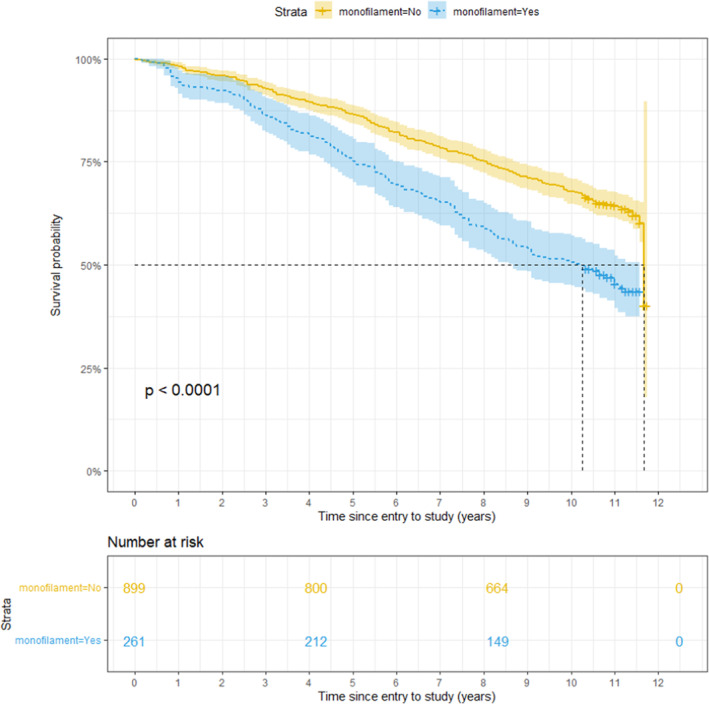
Survival probability over Time for subjects with Insensitivity to 10 g Monofilament (monofilament = Yes) and subjects without Insensitivity (monofilament = No). The *p*‐value is obtained after testing for a significant difference between the two groups with the log‐rank test.

**FIGURE 4:**
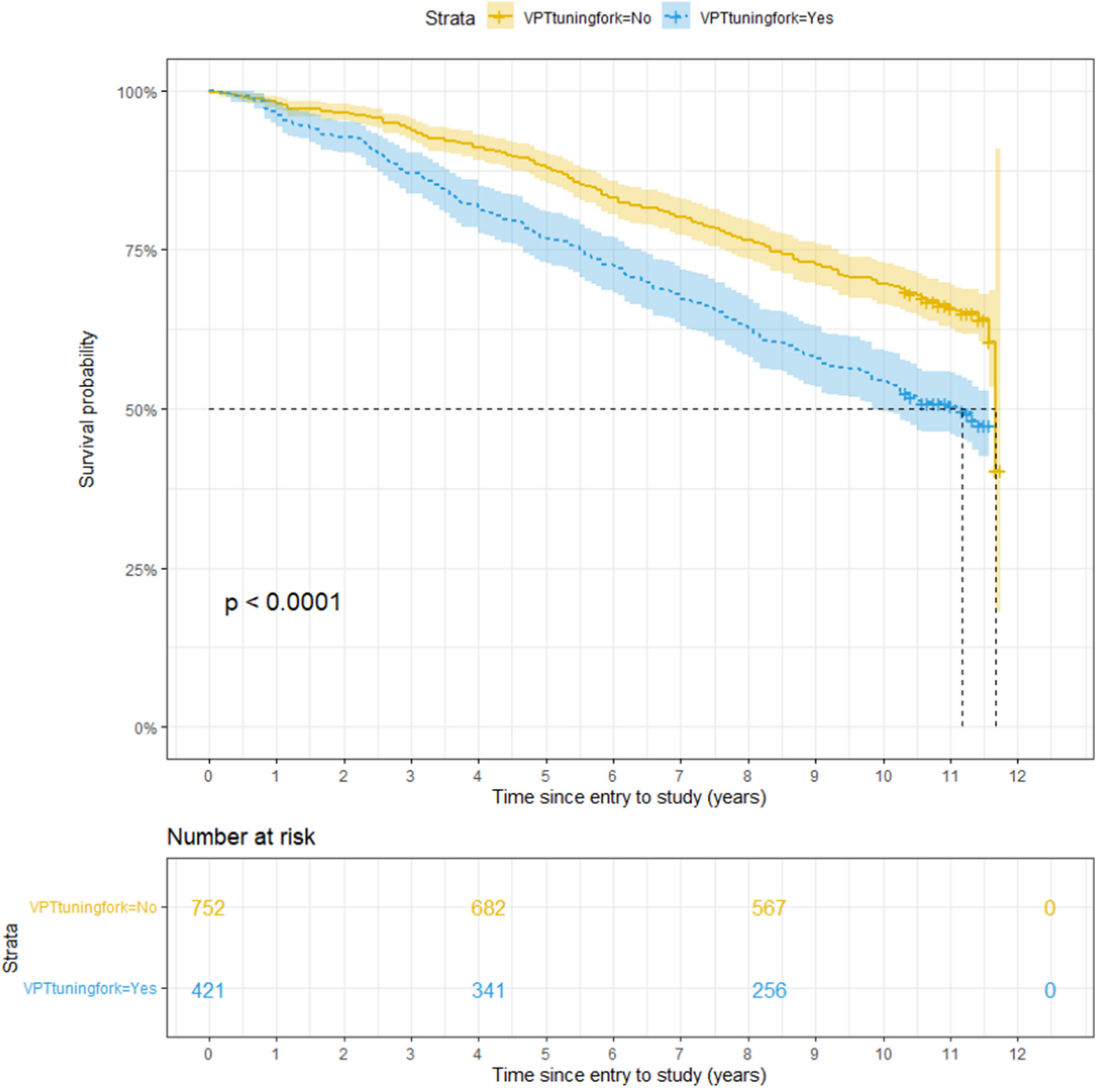
Survival probability over Time for subjects with Insensitivity to VPT tuning fork (VPTtuningfork = Yes) and subjects without Insensitivity (VPTtuningfork = No). The *p*‐value is obtained after testing for a significant difference between the two groups with the log‐rank test.

**FIGURE 5:**
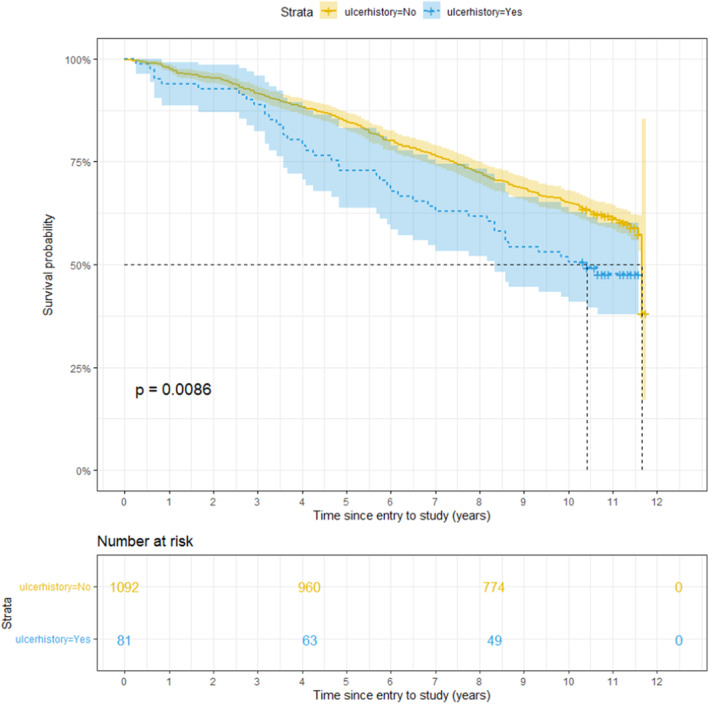
Survival probability over Time for subjects with previous ulcerations (ulcerhistory = Yes) and subjects without previous ulcerations (ulcerhistory = No). The *p*‐value is obtained after testing for a significant difference between the two groups with the log‐rank test.

Kaplan–Meier survival curves, with death as the outcome, show a statistically significant smaller survival probability for subjects that demonstrate insensitivity to a 10 g monofilament or a tuning fork and those with a history of foot ulceration. For those subjects, the survival probability decreases faster over time than for people who are not insensitive to a 10 g monofilament or tuning forks or have experienced a foot ulceration (Figures 3, 4, 5).

## 4 CONCLUSIONS

The risk factors for foot ulceration in this population identified by our multivariable survival analysis are consistent with validated prognostic models and clinical prediction rules for foot ulceration in international datasets, and in this study are shown to be sustained over the long term.^7^, ^11^ An inability to feel a 10 g monofilament or the vibration of a tuning fork underlines the central importance of neuropathy in the development of foot ulceration and their inclusion in the recommendations of diabetes clinical guidelines are justified.^3^, ^4^ Previous foot ulceration is well‐established as an independent predictor of foot ulceration risk, but it does signify advanced disease and is therefore of limited use in prevention.

This long‐term follow‐up predictive model differs from the original predictive model in that ankle brachial index, insulin use in the 3 months prior to recruitment, previous amputation and an ability to distinguish between cold and cool temperatures were not found to be independently predictive of foot ulceration in this new analysis.^10^ The increase in statistical power from the larger number of foot ulcers (from *n* = 23 to 77) has produced a predictive model with good discrimination; the C statistic (AUC) being 0.73 (95% CI: 0.674–0.805). This C statistic occupies the lower end of the confidence interval of the original predictive model (0.835 (95% CI 0.735 to 0.936)) and although there is no statistically significant difference between the two models, there may be residual confounding building up over the long follow‐up period.

Fifty‐three per cent of the original cohort who survived a further 10 years, gave consent to have their data collected and analysed. The observed long‐term incidence of foot ulceration of 18% in this cohort is consistent with foot ulcer incidence reported by others who have estimated the lifetime risk of foot ulceration in people with diabetes to be 25%.^20^ However, there is uncertainty about the true rate of ulceration in this cohort in the long term due to missing data for those who were deceased, lost to follow‐up or who did not provide consent for their long‐term outcomes to be collected. In any observational study of risk, there is a possibility that people will receive preventative interventions and the ulceration rate of this cohort may have been modified after general podiatric care.

By comparing demographic characteristics of those who died and those who survived and those who gave consent and those who did not, we have explored possible explanations for missingness. (Tables 5, 6, 7, 8) The profile of those who died compared with those who did not, shows those who died were older, had diabetes for longer and a greater number had a previous history of foot ulceration than those who survived and indicates the natural history of diabetes in this cohort population. (Tables 7 and 8).

**TABLE 5:** Demographic data for participants who were alive and consented to follow‐up.

Parameters (unit)	Statistic	Value
No. of DM patients	*N* (%)	311
Age (years)	Mean (SD)	66.7 (9.9)
Females	*N* (%)	160 (51.4%)
HbA1c	Mean (SD)	7.5 (1.5)
Time from diagnosis of diabetes (years)	Mean (SD)	9.1 (8.7)
Insensitivity to 10 g Monofilament	*N* (%)	45 (14.5%)
Insensitivity to VPT tuning fork	*N* (%)	91 (29.3%)
Previous history of ulceration	*N* (%)	19 (6.1%)
Previous history of amputation	*N* (%)	1 (0.3%)

*Note*: Demographic profiles at point of recruitment to original study 2006–2007.

**TABLE 6:** Demographic data for participants who were alive but did not consent to follow‐up.

Parameters (unit)	Statistic	Value
No. of DM patients	*N* (%)	277
Age (years)	Mean (SD)	68.0 (10.1)
Females	*N* (%)	173 (62.5%)
HbA1c	Mean (SD)	7.5 (1.4)
Time from diagnosis of diabetes (years)	Mean (SD)	7.4 (7.2)
Insensitivity to 10 g Monofilament	*N* (%)	48 (17.3%)
Insensitivity to VPT tuning fork	*N* (%)	77 (27.8%)
Previous history of ulceration	*N* (%)	12 (4.3%)
Previous history of amputation	*N* (%)	2 (0.7%)

*Note*: Demographic profiles at point of recruitment to original study 2006–2007.

**TABLE 7:** Demographic data of participants who died during the follow‐up and 10‐year foot ulceration were data available.

Parameters (unit)	Statistic	Value
No. of DM patients	*N* (%)	50
Age (years)	Mean (SD)	73.3 (8.1)
Females	*N* (%)	21 (42.0%)
HbA1c	Mean (SD)	7.2 (1.2)
Time from diagnosis of diabetes (years)	Mean (SD)	9.6 (7.5)
Insensitivity to 10 g Monofilament	*N* (%)	16 (32.0%)
Insensitivity to VPT tuning fork	*N* (%)	23 (46.0%)
Previous history of ulceration	*N* (%)	4 (8.0%)
Previous history of amputation	*N* (%)	1 (2.0%)

*Note*: Demographic profiles at point of recruitment to original study 2006–2007.

**TABLE 8:** Demographic data of participants who died during the follow‐up for whom 10‐year foot ulceration data were not available.

Parameters (unit)	Statistic	Value
No. of DM patients	*N* (%)	439
Age (years)	Mean (SD)	74.5 (8.4)
Females	*N* (%)	174 (39.6%)
HbA1c	Mean (SD)	7.5 (1.4)
Time from diagnosis of diabetes (years)	Mean (SD)	9.6 (9.1)
Insensitivity to 10 g Monofilament	*N* (%)	130 (29.6%)
Insensitivity to VPT tuning fork	*N* (%)	196 (44.6%)
Previous history of ulceration	*N* (%)	39 (8.9%)
Previous history of amputation	*N* (%)	10 (2.3%)

*Note*: Demographic profiles at point of recruitment to original study 2006–2007.

A comparison of the demographic profiles of those who gave consent to have their long‐term follow‐up data analysed and those who did not shows the consenters were slightly younger, had diabetes for longer, and more had a previous history of foot ulceration, concern about which may have acted as an incentive to participate in the research. (Tables 5 and 6) The reluctance of those who did not agree to the 10‐year follow‐up may stem from the fact fewer had experienced a foot ulcer and the study objectives may have been perceived to be less relevant to those individuals.

An important finding of this follow‐up study is the level of mortality (41%) at 10 years, a higher observed rate than that of foot ulceration. Age‐related mortality for Tayside populations as captured in the Scottish life expectancy tables for 2006–2008 in Tayside show that in the general population men aged 70 could expect to live for 13.42 years (95% CI 13.21 to 13.63) and women aged 70 for 15.56 years (95% CI 15.37 to 15.76).^21^ The people in this cohort exhibited a lower life expectancy than the general Scottish population. Unfortunately, we did not have any information about the cause of death for those who died and future research should seek to obtain this information.

Our analyses also found that those people who exhibited risk factors for foot ulceration such as an inability to feel a 10 g monofilament or vibration from a tuning fork or a history of foot ulceration demonstrated shorter survival and life expectancy. It seems reasonable to suppose that these risk factors are indicative of more systemic complications of diabetes such as cardiovascular disease and further research is required to understand whether targeted interventions to manage cardiovascular risk can reduce mortality as well as foot ulceration.

### 4.1 The strengths and weaknesses of this research

Cohort studies to identify risk factors for diabetes‐related foot ulcerations over the long‐term are rare and this follow‐up study reveals a higher mortality than would be expected in the general population and an incidence of foot ulceration of 18% after 10 years.

The accuracy of the estimates is threatened by the administrative policy of destroying the podiatry records of people who were registered with the NHS Tayside podiatry service once they are deceased. This prevented the ascertainment of foot ulcers for the majority of those who did not survive. For the 116 people who were lost to follow‐up because their SCI Diabetes electronic record was no longer available, possibly due to them no longer living in Scotland, these missing data may also be a source of underestimation of foot ulceration. (Table 9).

**TABLE 9:** Demographic characteristics of participants who were lost to follow‐up for whom 10‐year foot ulceration data are not available.

Parameters (unit)	Statistic	Value
No. of DM patients	*N* (%)	116
Age (years)	Mean (SD)	70.8 (10.1)
Females	*N* (%)	53 (45.7%)
HbA1c	Mean (SD)	7.8 (2.0)
Time from diagnosis of diabetes (years)	Mean (SD)	8.6 (7.8)
Insensitivity to 10 g Monofilament	*N* (%)	27 (23.3%)
Insensitivity to VPT tuning fork	*N* (%)	40 (34.5%)
Previous history of ulceration	*N* (%)	8 (6.9%)
Previous history of amputation	*N* (%)	3 (2.6%)

*Note*: Demographic profiles at point of recruitment to original study 2006–2007.

By analysing the original results of the diagnostic tests, symptoms and signs observed in people with diabetes who took part in survival analyses with death as the outcome, we have shown that those who exhibit an inability to feel a 10 g monofilament, the vibrations of a tuning fork or a history of foot ulceration had a shorter survival than those who did not.

## AUTHOR CONTRIBUTIONS

**Shijat Ali Mohammed:** Formal analysis (supporting); project administration (supporting); visualization (equal); writing – original draft (supporting); writing – review and editing (supporting). **Fay Crawford:** Conceptualization (equal); data curation (lead); funding acquisition (lead); investigation (equal); methodology (supporting); project administration (equal); resources (equal); supervision (supporting); validation (equal); visualization (equal); writing – original draft (lead); writing – review and editing (lead). **Genevieve Isabelle Cezard:** Investigation (supporting); methodology (supporting); supervision (supporting); validation (equal); visualization (equal); writing – original draft (supporting); writing – review and editing (supporting). **Michail Papathomas:** Conceptualization (equal); formal analysis (lead); investigation (equal); methodology (lead); resources (equal); software (lead); supervision (lead); validation (equal); visualization (equal); writing – original draft (supporting); writing – review and editing (supporting).

## FUNDING INFORMATION

This work was funded as part of a wider project by the National Institute for Health Research (NIHR) Health Technology Assessment (HTA) Programme (HTA project: 15/171/01). The views expressed are those of the authors and not necessarily those of the NIHR or UK Department of Health and Social Care.

## CONFLICT OF INTEREST STATEMENT

The authors have no conflicts of interest.

## ETHICS STATEMENT

Original favourable opinion from Tayside Committee on Medical Research Ethics A (REC number 04/S1401/197). For the 10‐year follow‐up, a favourable opinion was received from Scotland A Research Ethics Committee [REC reference 16/SS/0213] and Caldicott approval obtained from NHS Tayside [Reference number IGTCAL3842] and R&D approval obtained from the sponsor (NHS Fife [Reference 17–01497542]). The study was registered on the BioMed Central ISRCTN clinical trial register [Reference number 10550720].

## CONSENT FOR PUBLICATION

All authors agree to the publication of this manuscript.

## Supporting information


Appendix S1

Appendix S2

## Data Availability

All data generated or analyses conducted during the current study are available from the corresponding author.

## References

[1] International Diabetes Federation IDF Diabetes Atlas . 10th edn. Brussels, Belgium. 2021. Accessed October 29, 2023. https://www.diabetesatlas.org

[2] Kerr M , Barron E , Chadwick P , et al. The cost of diabetic foot ulcers and amputations to the National Health Service in England. Diabet Med. 2019;36:995‐1002. 31004370 10.1111/dme.13973

[3] NICE NG19 . Diabetic Foot Problems Prevention and Management. Accessed October 29, 2023. https://www.nice.org.uk/guidance/ng19 32045177

[4] International Working Group of the Diabetic Foot (IWGDF) . Accessed October 29, 2023. https://iwgdfguidelines.org/

[5] Beulens JWJ , Yauw JS , Elders PJM , et al. Prognostic models for predicting the risk of foot ulcer or amputation in people with type 2 diabetes: a systematic review and external validation study. Diabetologia. 2021;64:1550‐1562. 33904946 10.1007/s00125-021-05448-w PMC8075833

[6] Monteiro‐Soares M , Ribas R , Pereira da Silva C , et al. Diabetic foot ulcer development risk classifications' validation: a multicentre prospective cohort study. Diabet Res. Clin. Pract. 2017;127:105‐114. 10.1016/j.diabres.2017.02.034 28340359

[7] Crawford F , Cezard G , Chappell FM , et al. A systematic review and individual patient data meta‐analysis of prognostic factors for foot ulceration in people with diabetes: the international research collaboration for the prediction of diabetic foot ulcerations (PODUS). Health Technol Assess. 2015;19:1‐210. 10.3310/hta19570 PMC4781379 26211920

[8] Heggie R , Chappell F , Crawford F , et al. Complication rate among people with diabetes at low risk of foot ulceration in fife, UK: an analysis of routinely collected data. Diabet Med. 2020;37:2116‐2123. 32510602 10.1111/dme.14339

[9] Walsh JW , Hoffstad OJ , Sullivan MO , Margolis DJ . Association of diabetic foot ulcer and death in a population‐based cohort from the United Kingdom. Diabet Med. 2016;33:1493‐1498. 26666583 10.1111/dme.13054

[10] Crawford F , McCowan C , Dimitrov BD , et al. The risk of foot ulceration in people with diabetes screened in community settings: findings from a cohort study. QJM. 2011;104:403‐410. 21186178 10.1093/qjmed/hcq227

[11] Crawford F , Chappell FM , Lewsey J , et al. Risk assessments and structured care interventions for prevention of foot ulceration in diabetes: development and validation of a prognostic model. Health Technol Assess. 2020;24:1‐198. 10.3310/hta24620 PMC7768791 33236718

[12] Steyerberg EW . Selection of Main effects. Clinical Prediction Models: A Practical Approach to Development, Validation, and Updating. Springer; 2009:191‐211.

[13] Harrell FE . Regression Modelling Strategies: with Applications to Linear Models, Logistic Regression, and Survival Analysis. Springer‐Verlag; 2001.

[14] Essential Medical Statistics , ed. Eds Kirkwood and Sterne. 2nd ed. Wiley‐Blackwell; 2003.

[15] Riley RD , Snell KI , Ensor J , et al. Minimum sample size for developing a multivariable prediction model: PART II – binary and time‐to event outcomes. Stat Med. 2019;38(7):1276‐1296. 30357870 10.1002/sim.7992 PMC6519266

[16] Chambless L , Diao G . Estimation of time‐dependent area under the ROC curve for long term risk prediction. Stat Med. 2006;25(20):3474‐3486. 16220486 10.1002/sim.2299

[17] Zhang MJ , Zhang X , Scheike TH . Modeling cumulative incidence function for competing risks data. Expert Rev Clin Pharmacol. 2008;1(3):391‐400. 19829754 10.1586/17512433.1.3.391 PMC2760993

[18] Zhang Z . Survival analysis in the presence of competing risks. Ann Transl Med. 2017;5:47. 28251126 10.21037/atm.2016.08.62 PMC5326634

[19] Collett D . Modelling survival data in medical research. CRC press; 2023.

[20] Singh N , Armstrong DG , Lipsky BA . Preventing foot ulcers in patients with diabetes. JAMA. 2005;293:217‐228. 15644549 10.1001/jama.293.2.217

[21] Life Expectancy in Scottish Areas . National Registers of Scotland. Accessed October 29, 2023. https://www.nrscotland.gov.uk/statistics‐and‐data/statistics/statistics‐by‐theme/life‐expectancy/life‐expectancy‐in‐scottish‐areas

