# Supplementary material for: The 10‐year follow‐up of a community‐based cohort of people with diabetes: The incidence of foot ulceration and death
Source: Endocrinol Diabetes Metab. 2023 Nov 21;7(1):e459. doi: 10.1002/edm2.459 (PMC10782183; doi:10.1002/edm2.459)
Supplement: Supplementary file 1 — Appendix S1 [file EDM2-7-e459-s001.docx]

**Supplementary files: Additional information on fitted Cox proportional hazards model .**

We provide additional information on the fitted Cox proportional hazards model shown on Section ‘Cox proportional hazards’ of the main manuscript, relevant to making predictions. Specifically, we provide below different values for the cumulative hazard function H_0_(t).

| **Cumulative Baseline Hazard (H0)** | **time (t) in years** |
| --- | --- |
| 0.003 | 0.5 |
| 0.005 | 1.0 |
| 0.006 | 1.5 |
| 0.006 | 2.0 |
| 0.007 | 2.5 |
| 0.007 | 3.0 |
| 0.007 | 3.5 |
| 0.009 | 4.0 |
| 0.009 | 4.5 |
| 0.011 | 5.0 |
| 0.012 | 5.5 |
| 0.014 | 6.0 |
| 0.015 | 6.5 |
| 0.018 | 7.0 |
| 0.020 | 7.5 |
| 0.022 | 8.0 |
| 0.025 | 8.5 |
| 0.028 | 9.0 |
| 0.036 | 9.5 |
| 0.039 | 10.0 |
